# Hackathons as Stepping Stones in Health Care Innovation: Case Study With Systematic Recommendations

**DOI:** 10.2196/17004

**Published:** 2020-03-24

**Authors:** Akira-Sebastian Poncette, Pablo-David Rojas, Joscha Hofferbert, Alvaro Valera Sosa, Felix Balzer, Katarina Braune

**Affiliations:** 1 Einstein Center Digital Future Berlin Germany; 2 Department of Anesthesiology and Intensive Care Medicine Charité – Universitätsmedizin Berlin (corporate member of Freie Universität Berlin, Humboldt-Universität zu Berlin, and Berlin Institute of Health) Berlin Germany; 3 Hacking Health Berlin Germany; 4 Department of Architecture in Health Berlin Institute of Technology Berlin Germany; 5 Department of Pediatric Endocrinology and Diabetes Charité – Universitätsmedizin Berlin (corporate member of Freie Universität Berlin, Humboldt-Universität zu Berlin, and Berlin Institute of Health) Berlin Germany

**Keywords:** digital health, transdisciplinary research, hackathon, technological innovation, patient-centered care, social media

## Abstract

**Background:**

Until recently, developing health technologies was time-consuming and expensive, and often involved patients, doctors, and other health care professionals only as passive recipients of the end product. So far, users have been minimally involved in the ideation and creation stages of digital health technologies. In order to best address users’ unmet needs, a transdisciplinary and user-led approach, involving cocreation and direct user feedback, is required. In this context, hackathon events have become increasingly popular in generating enthusiasm for user-centered innovation.

**Objective:**

This case study describes preparatory steps and the performance of a health hackathon directly involving patients and health care professionals at all stages. Feasibility and outcomes were assessed, leading to the development of systematic recommendations for future hackathons as a vehicle for bottom-up innovation in health care.

**Methods:**

A 2-day hackathon was conducted in February 2017 in Berlin, Germany. Data were collected through a field study. Collected field notes were subsequently discussed in 15 informal meetings among the research team. Experiences of conducting two further hackathons in December 2017 and November 2018 were included.

**Results:**

In total, 30 participants took part, with 63% (19/30) of participants between 25 and 34 years of age, 30% (9/30) between 35 and 44 years of age, and 7% (2/30) younger than 25 years of age. A total of 43% (13/30) of the participants were female. The participation rate of medical experts, including patients and health care professionals, was 30% (9/30). Five multidisciplinary teams were formed and each tackled a specific health care problem. All presented projects were apps: a chatbot for skin cancer recognition, an augmented reality exposure-based therapy (eg, for arachnophobia), an app for medical neighborhood connectivity, a doctor appointment platform, and a self-care app for people suffering from depression. Patients and health care professionals initiated all of the projects. Conducting the hackathon resulted in significant growth of the digital health community of Berlin and was followed up by larger hackathons. Systematic recommendations for conducting cost-efficient hackathons (n≤30) were developed, including aspects of community building, stakeholder engagement, mentoring, themes, announcements, follow-up, and timing for each step.

**Conclusions:**

This study shows that hackathons are effective in bringing innovation to health care and are more cost- and time-efficient and potentially more sustainable than traditional medical device and digital product development. Our systematic recommendations can be useful to other individuals and organizations that want to establish user-led innovation in academic hospitals by conducting transdisciplinary hackathons.

## Introduction

Research in health care is expensive, time-consuming, and does not always ensure the development and implementation of sustainable and appropriate technologies that best address the needs and requirements of patients, doctors, and health care professionals [1]. A transdisciplinary approach, together with direct end-user feedback, may benefit the cost-efficient development of innovative health technologies [2]. In this context, hackathons have become an increasingly popular venue for health care institutions to generate enthusiasm for innovation.

The term *hackathon* derives from the words *hack* and *marathon*. In this context, *hacking* refers to intensive collaborative computer programming. Since the late 1990s, the concept of gathering experts into teams to foster collaboration and solve pressing problems has become increasingly popular. Initially, hackathons were highly targeted at those working in tech [3] but, more recently, hackathons have also found a niche in the medical field and academic literature [4]. Since 2010, hundreds of health hackathons have been documented worldwide, with most of them being held in the United States [5].

The question remains whether hackathons are an effective method to accelerate the creation of novel medical technology. The Consortium for Affordable Medical Technologies (CAMTech), based at the Massachusetts General Hospital’s Global Health department, recently published the outcomes of 12 hackathons from 2012 to 2015 in India, Uganda, and the United States [6]. The projects initiated through these events were often followed up afterward and have reached pilot-testing stages, started clinical trials, or even resulted in the formation of new companies. The health hackathon model, including preceding priming activities and targeted postevent support, were rated as a reliable source of solutions to challenges in health care.

However, these results mainly speak for hackathons organized either in the United States or by people from the United States. Research on hackathons in Europe is rare or almost nonexistent. A possible reason may be that the organization of a hackathon requires considerable effort and costs, especially for organizations without any previous experience in this area, such as academic hospitals [7]. Furthermore, hackathons in Europe, especially in Germany, are relatively recent and unknown to most health care providers and operators.

This case study analyzed the preparation and performance of a transdisciplinary health hackathon conducted in Berlin, Germany. Feasibility and outcomes were assessed, leading to the development of systematic recommendations for future health hackathons.

## Methods

### Study Setting

The 2-day hackathon was conducted over a weekend in February 2017 in Berlin, Germany. The organizing team consisted of 10 people: a core organizing team of three people (ASP, AVS, and JH) and seven volunteers. All members of the organizing team were members of the nonprofit organization Hacking Health.

### Hackathon Resources

Our first most valuable resource was a nonprofit organization called Hacking Health, founded and operated in Montreal, Canada, since 2012 [8]. Hacking Health has organized health hackathons worldwide, mostly across Canada, the United States, the Netherlands, France, and eventually in Germany in 2017 with the hackathon presented here.

Prior to this event, the German chapter of Hacking Health was founded by three volunteers in Berlin. Supported by Hacking Health Canada, they were provided with all relevant resources for building a successful digital health community free of charge. The newly founded Hacking Health Berlin chapter then organized various local events, from workshops about biomedical technology to talks about digital health. Since then, the chapter and its network have grown gradually in Berlin [9].

A further helpful resource was the Health Hackathon Handbook by MIT Hacking Medicine, which is available online for free [10] and was used as a guide for organizing the hackathon studied here. Founded at the Massachusetts Institute of Technology (MIT) in Boston, USA, MIT Hacking Medicine aims at accelerating medical innovation by carrying out health hackathons, workshops, or networking gatherings all over the world [11].

### Hackathon Preparation

The theme of the hackathon was chosen to be specific, on the one hand, to target physicians and other health care professionals and, on the other hand, to be as broad as possible to include participants from any professional background. By choosing the theme *Patient Care Goes Digital*, we intentionally focused on digital health and patient-centered care, thereby excluding the fitness and lifestyle sector.

In order to reach a wide multidisciplinary community that included medical professionals and patients, we released targeted announcements 10 weeks prior to the event via social networks: Facebook, Meetup, and Twitter. Additionally, leading senior physicians and several resident physicians were contacted personally. In promoting the event, the general concept of hackathons was explained as an “open transdisciplinary workshop,” since many potential participants—especially from the medical sector—were still unfamiliar with the term *hackathon*. Additionally, 3 weeks prior to the hackathon, a prehackathon event was organized as an introduction, which was not mandatory for participation at the hackathon.

To participate, applicants were asked to fill out an online questionnaire and provide a brief description of their motivation to participate, their background, and their skill set using a Web-based, team-building platform called *Sparkboard* [12]. Team building was started online 2 weeks prior to the hackathon and was moderated by a member from the organizing team. Participants were encouraged to upload project ideas or *pain points* they would like to work on and develop solutions for onto Sparkboard in advance. A solution approach was not mandatory.

### Hackathon Event

The hackathon event was started on a Saturday morning and opened with an inspirational keynote speech on the dynamics of digital health and instructions about hackathon-related practices, in order to increase participants’ understanding of the complexity of health care-related challenges. The majority of participants, especially physicians and health care professionals, had little previous experience with digital health- or innovation-related events and were used to more rigidly structured organizations and practices. After the opening keynote speech, participants were given the opportunity to pitch their project idea within 1 minute and find members for their team with the right skill set to help in developing the solution. Most of the hackathon was then spent on further developing ideas and prototypes (ie, *hacking*) and on preparing the final pitch and demonstration.

Throughout the event, all teams were supported by mentors from the local academic hospital Charité−Universitätsmedizin Berlin (doctors and psychologists), the Berlin Institute of Technology (engineers, developers, and architects), the software company ThoughtWorks (developers), and the Hasso-Plattner-Institute for Design Thinking (designers), as well as by patients living with chronic conditions and entrepreneurs from Berlin-based health care startups. Exchange of expertise and assistance was encouraged between teams.

On Sunday afternoon, the hackathon concluded with a public pitch session lasting 5 minutes each. An expert jury panel voted to select two winning teams. The multidisciplinary jury consisted of five members, including a physician with an entrepreneurial background, a designer, two developers, and a patient with a chronic condition. The jury criteria included the following:

1. Innovation potential and feasibility of the idea.
2. Execution of the idea at the hackathon.
3. Multidisciplinary composition of the team.
4. Design and user experience of the prototype.
5. Presentation of the project.

Instead of awarding monetary prizes, we collaborated with Berlin’s leading design-thinking studios to enable the winning teams to continue their project work supported by a team of experts and design-thinking workshops, of which both teams took part in about 3 months after the hackathon.

### Data Collection and Analysis

Data was collected through a field study in the context of the hackathon event in February 2017. Collected field notes were subsequently discussed during informal meetings within the research team between February 2017 and February 2019. Additionally, experiences of conducting two further hackathons in December 2017 and November 2018 were included. All authors were involved in either one, two, or all three hackathons. In total, 15 meetings were held, with the goals to develop systematic recommendations for conducting a health hackathon on the basis of this presented hackathon and to examine the impact of a smaller, cost-efficient hackathon on the digital health community of Berlin as a large city.

The research team consisted of a resident anesthesiologist with expertise in intensive care medicine, geriatrics, and digital health (ASP); a resident pediatric endocrinologist and patient advocate with expertise in digital health and patient-centric care (KB); a health care entrepreneur with a professional background in psychology (JH); an architect with training in evidence-based design research for health care (AVS); a professor for digital health, who is a consultant anesthesiologist and computer scientist (FB); and a microbiologist trained in human-centered design and innovation (PDR). Assumptions were consistently challenged in the transdisciplinary team setup.

## Results

### Hackathon Demographics and Projects

In total, 30 participants took part in the hackathon in February 2017. Their age varied across different groups, with 63% (19/30) of attendees between 25 and 34 years of age, 7% (2/30) younger than 25 years of age, and 30% (9/30) between 35 and 44 years of age. A total of 43% (13/30) of our participants were female. The participation rate of medical experts—physicians and health care professionals, as well as patients as experts for their disease management in everyday life—was 30% (9/30); the majority of the remaining participants were developers and designers.

Prior to the hackathon, 14 projects were uploaded to Sparkboard by participants to sign up and form teams. The jury selected five projects based on the previously established criteria, which applied later on at the hackathon. The teams consisted of 6 members each with a multidisciplinary background. All presented projects were mobile apps: a chatbot for skin cancer recognition; a mobile phone-powered, augmented reality exposure-based therapy (eg, for patients suffering from arachnophobia); an app for medical neighborhood connectivity to improve the experience of patients who have to visit several doctors; a doctor appointment platform; and a self-care app for patients suffering from depression. Patients, physicians, or other health care professionals initiated all of the projects.

### Hackathon Flow

**Figure 1:**
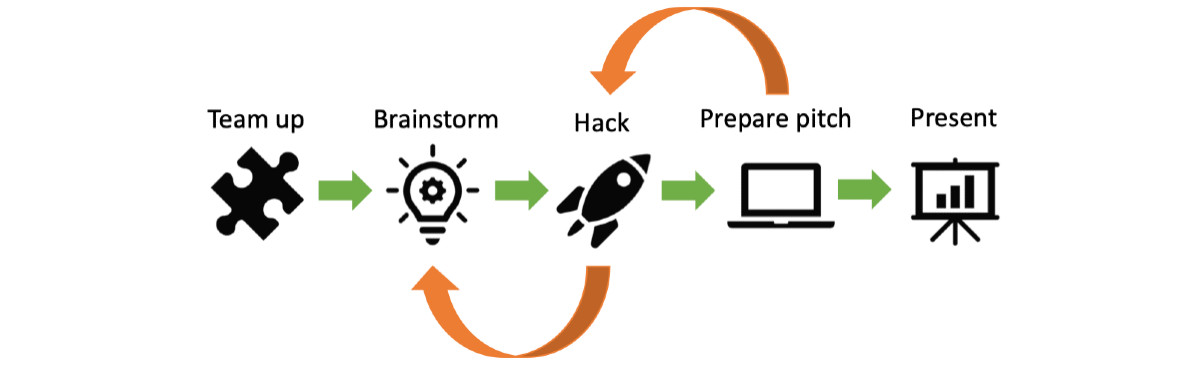
Hackathon flow, from teaming up to demonstration of the project pitch and working prototype (green arrows). In contrast to our expectations, teams often jumped back and forth between brainstorming, hacking, and preparing the pitch (orange arrows).

After multidisciplinary team formation, project ideas were discussed in brainstorm sessions within the teams, often involving techniques using post-it notes (eg, Venn diagrams) [13]. During this phase, mentors with medical expertise were recruited to explore the pain point and solution approach in relation to several health care actors (eg, physician, nurse, or patients). Afterward, participants carried on with their *hacking* and explored solution approaches with the support of information technology- or design-focused mentors. As a result of this iterative process, the teams developed prototypes (eg, a software mock-up) demonstrating their ideas and solutions. Often in parallel to hacking, one of the team members started working on their pitch. Finally, solutions were presented at a plenum including the jury, ideally showing a working prototype. As sketched in Figure 1, in contrast to our expectations, teams often moved back and forth between brainstorming, hacking, and preparing the pitch.

### Performance of the First Hackathon

Following this hackathon with 30 participants in February 2017, the Berlin Institute of Health (BIH), a scientific institution for translation and precision medicine, partially funded further hackathons in December 2017 and November 2018. Both larger hackathons included about 75 active participants each, whereas more than 300 participants took part in the opening and closing ceremonies. All three hackathons were unique, self-contained events.

**Figure 2:**
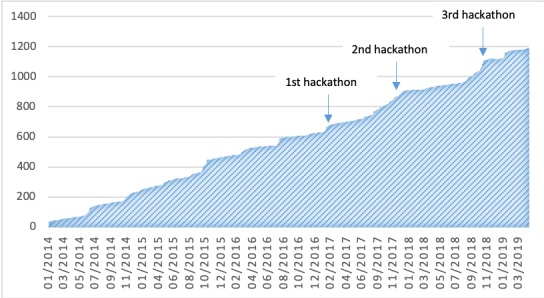
Total number of members of the Meetup group Hacking Health Berlin from January 2014 to March 2019. The three hackathons are marked with arrows [9].

Regarding impact on the digital health community of Berlin, relevant growth of the Meetup group Hacking Health Berlin could be recorded, especially before the hackathons (see Figure 2) [9]. The average growth of the community is 0.6 (SD 1.4) new members per day. A total of 4 weeks prior and 2 weeks after the first, second, and third hackathons, the average growth was 1.2 (SD 2.0), 1.3 (SD 1.2), and 2.0 (SD 2.6) new members per day, respectively.

### Systematic Recommendation for Conducting a Cost-Efficient Health Hackathon

#### Overview

**Figure 3:**
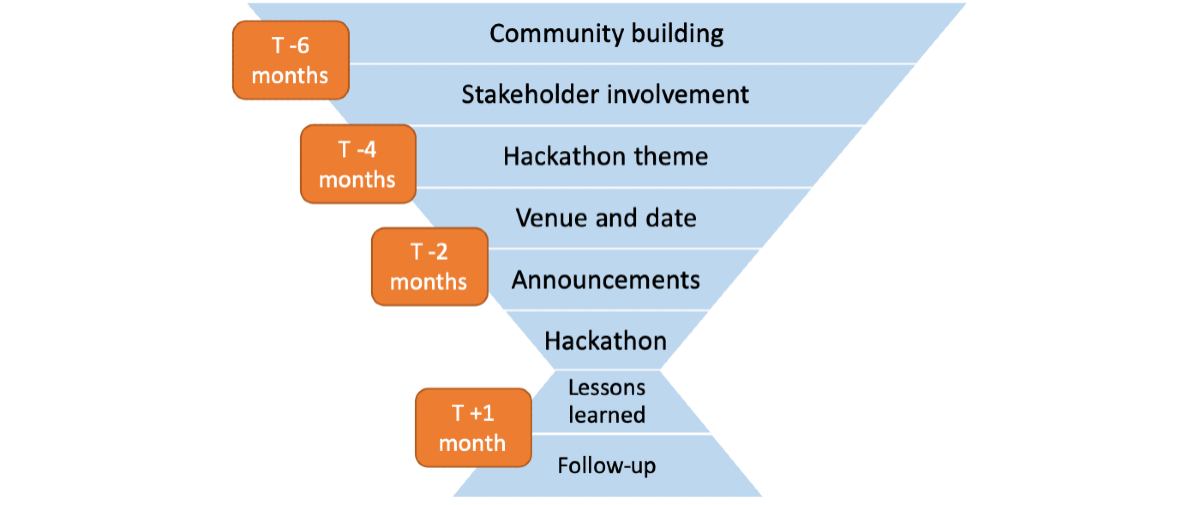
Systematic recommendation for conducting a cost-efficient hackathon with 30 or fewer participants. The time (T) to action is visualized in orange boxes. The hackathon occurs at T0.

The developed systematic recommendation includes eight steps (see Figure 3). In preparation for the hackathon, five steps have to be undertaken (ie, community building, stakeholder involvement, selection of the hackathon theme, venue and date, and announcements); after the hackathon, two steps are recommended (ie, lessons learned and follow-up). The timing for each step was chosen to work for the organization of a hackathon with no more than 30 participants; depending on the available community, the preparation of larger hackathons (ie, >100 participants) should be started at least 2 months earlier. In our experience, smaller hackathons are much more cost-efficient, with up to €100 per capita, compared to larger events with approximately €1000 spent per capita.

#### Community Building and Stakeholder Involvement

The intention or drive to conduct a hackathon derives from the interest among a specific group of people. An existing community of at least 100 people is recommended. In this context, the community is defined as a stable and loyal network of people in an online or offline context, hence, potential participants that can be contacted online or addressed at related networking gatherings. Strategies to grow a digital health community include organizing regular small events or gatherings, advertising these events locally and on social media, and building up a member database (eg, using mailing lists or social media groups). This step should be achieved at least 6 months prior to the hackathon.

A successful hackathon also depends on stakeholder and expert involvement, people who are ideally members of the organizing team. However, senior physicians or developers might be unable to commit to organizing or participating in an entire hackathon [14]. Therefore, the participation of experienced advisors and mentors is highly recommended and their recruitment should be performed well in advance, due to their profession-related constraints. The establishment of a local network may reduce personnel expenses, as mentors, keynote speakers, or jury members often provide their support free of charge and do not have to travel far in order to attend the event. In particular, these volunteers might be more attracted to smaller *bootstrapping* events than larger events with a more corporate character.

A local network is essential for the acquisition of financial resources. In our experience, the time spent cold-contacting potential sponsors should be better invested in the growth of the network. Rewarding options for sponsors may include advertising banners at the event, the opportunity to present as a keynote speaker, challenge cocreation, or branded giveaways to participants; sponsors may even send recruiters to the event to scout talent. In our opinion, it has proven to be helpful to create a brochure for potential sponsors explaining different sponsorship options and *packages* (eg, platinum, gold, or silver sponsorship).

#### Hackathon Theme

The hackathon theme is an essential selling point to medical professionals and patients. Along these lines, the theme of a hackathon, especially when held for the first time, should be general enough to attract a broad spectrum of potential participants. Following hackathons may be more specific to certain medical or problem areas. In both cases, it is important to highlight how a health-related hackathon may be different from hackathons that are more general or more specific to other subject areas.

When selecting a theme, it is important that the corresponding expertise—ideally provided by a patient or practicing physician—is represented by at least one expert as part of the organizing team. Once a theme is chosen, specific challenges should be defined, matching the theme. We recommend three to five challenges in total. As an alternative to a specific theme, only challenges with different themes may be chosen. In this case, we recommend no more than three challenges.

#### Venue and Timing

In contrast to larger hackathons, it is much easier to find a fitting venue for smaller events (n≤30), as only a larger room and an *escape room* are needed. When choosing the venue, factors such as light, air quality, and acoustics should be of importance, taking into account that participants are spending most of their time in that venue, mostly in teams sitting at tables. Choosing a big room for many teams has the advantage of fostering team spirit. However, especially at the beginning of the hackathon where conversation is most important, it can get very noisy. Smaller escape rooms for quick team meetings might solve this problem.

Event organization for a hackathon is mostly similar to organizing conferences. However, we observed three major differences. Firstly, we recommend leaving it to the participants when to stop working on their projects in the evening. Hence, a warm snack should be prepared at night for teams who prefer to stay longer. Secondly, from our experience, intense collaborative work burns lots of calories and participants usually consume more food than average conference attendees. Thus, a warm lunch and dinner should be prepared in a sufficient quantity, instead of small snacks or cold dishes only. Lastly, a snack bar should be ready at all times with fruits, nuts, vegetables, coffee, tea, juice, and water. We recommend hiring a professional caterer for events of 50 people and above. Alternatively, more affordable options such as local delivery services may be used for smaller hackathons instead.

#### Announcements and Call for Application

Depending on the size of the hackathon, announcements for application or registration should be made 2-4 months prior to the event via all available channels, including social media (eg, Twitter, LinkedIn, Meetup, and Facebook), hospitals’ internal blackboards, mailing lists, and cold emails to various stakeholders, which may be shared among their staff. Recurrent announcements may be biweekly, more condensed before deadlines, and justifiable through new information (eg, new challenges and partners) or approaching deadlines for applications. For larger hackathons, it has proven successful to extend the application deadline at least once, with the first deadline being 2 weeks prior to the event.

In contrast to conferences, a successful hackathon highly depends on the quality of participants, their preparation, and their composition (eg, equally represented professions). Although it is time-consuming, it is advisable to select the participants individually on the basis of a short questionnaire and to ensure that all disciplines and skill sets are represented sufficiently. The following participants should be selected, in order to include the desired disciplines: practicing physicians or health care professionals; developers, engineers, and designers, with further detailed descriptions of their skills, especially for the developers; patients, with descriptions of their conditions and experience (eg, in advocacy); and other professionals, including architects, other researchers, and business economists.

To break the ice, it is important to inform potential participants about what a hackathon actually is. Moreover, the word *hack* has a negative connotation to several individuals. One way to prevent this would be to use an alternative word such as *datathon* or *collaborathon* instead [15].

#### Hackathon

Whereas a hackathon often starts and ends with long opening and closing ceremonies that include keynote speeches on recent challenges in health care, panel discussions, or workshops, these activities are optional. Factors that significantly improve the outcome of a hackathon are as follows:

1. The acknowledgement of the expertise of the participants.
2. The presentation of the hackathon challenges.
3. The introduction of mentors.
4. The incitement of team spirit.

At a hackathon, participants are eager to solve entrenching problems, hence, less than 30 minutes for the welcoming remarks, an opening keynote speech, and introduction of the challenges is sufficient. Following the introduction, all team leaders should pitch their project, without using slides, in 1 minute. To keep within the timeline, an audio signal (eg, playing a jingle) interrupting the pitch after the time is up has been proven helpful (see Table 1).

Team building should be initiated by the organizers prior to the event, depending on team constellations and their multidisciplinary backgrounds. By starting prior to the event, optimal team compositions can be achieved, and most of the time can be spent on hacking. Mentors should fill in a profile that can be presented to the teams in order to find the right mentor faster, and mentors should be given acknowledgment. Occasionally, teams might need a space to think and work in silence. This can be achieved by providing teams with “do not disturb” signs. For relaxation, activities like yoga or meditation may be offered.

Either on Saturday evening or Sunday morning, participants may be given the opportunity to get feedback from a professional pitching expert. Each team should get at least 10 minutes of mentoring time, including the 3-minute final pitch.

**Table 1:** Example schedule of a 2-day weekend hackathon.

Time of day		Schedule items and events	
		Day 1: Saturday	Day 2: Sunday
**Morning**			
	07:00		Coffee and breakfast
	08:00		Pitch clinic (10 minutes for each team)
	08:30	Registration opening Coffee and breakfast	
	09:30	Welcome	
	09:40	Keynote speech	Hacking and mentoring
	09:50	Introduction of the challenges	
	10:00	60-second pitches (no slides)	
	10:15	Final team formation^a^	
	10:30	Teams will be assigned hacking space	
	11:00	Hacking and mentoring	
**Noon**			
	12:15		Drinks and canapés^b^
	12:30	Lunch	
	12:45		Group photo
**Afternoon**			
	13:00	Hacking and mentoring	
	14:00	Yoga session	Submission deadline
	14:05		Jury briefing
	14:40		Keynote speech^b^
	15:00	Hacking and mentoring	Public pitching of demos (3 minutes each with 2 minutes of Q&A)^b^
	17:30		Drinks and canapés^b^
**Evening and night**			
	18:00	Dinner	Announcement of winners with award ceremony^b^
	19:00	Hacking	After-hours drinks and networking^b^
	21:00	Optional hacking through the night	
	00:00	Midnight snack	

^a^Team formation may also be started online 2 weeks prior to the event.

^b^These events are open to the public, while all others are only open to active hackathon participants and mentors.

#### Lessons Learned and Follow-Up

A debriefing meeting among the organizing team a few days after the hackathon should be planned in advance to discuss and document optimization potential for future hackathons.

To improve the performance of the hackathon, a survey should be handed out in person and sent to the participants after the hackathon [16]. Questions may include demographic data such as age or professional background of the participants, feedback on the hackathon, as well as rating scores representing their levels of confidence in starting a health care project before and after the hackathon. To ensure sufficient return, the survey can be combined with incentives, such as access to videos and photos of the hackathon or a discount to future hackathons or other events. Participants should be followed up 3, 6, and 12 months after the event by email (eg, with a survey) to determine whether the teams have continued working on their projects.

## Discussion

### Principal Findings

This study describes and analyzes a small and cost-efficient health hackathon with 30 participants. In total, five multidisciplinary teams were formed, and each team tackled a specific health care problem. Performing the hackathon resulted in a significant growth of the digital health community in Berlin and the execution of subsequent larger hackathons.

By including the results from the subsequent larger hackathons, a systematic recommendation for conducting cost-efficient hackathons (n≤30) was developed. These recommendations include aspects of community building, hackathon theme, announcements, and timing for each step.

### Hackathon Performance

Compared with other health hackathons, the final number of participants of our health hackathon was smaller [5]. We purposefully limited the number of participants, subsequently reducing logistic tasks, costs, and workload for organizing staff.

From our point of view, the greatest potential and value of hackathons lies in providing an opportunity for people to meet and collaborate throughout the event and at mid- to long-term time points after the event. A hackathon puts experts’ brains into the right gear and inspires them to think in an unconventional fashion. This does not necessarily have to result in a prototype immediately; however, it may be achievable in the near future. The impact of a hackathon has been described in various ways [12,13]. Silver et al pointed out that the best metric for impact is to measure how many teams continue to work on their solution after the event [12]. Other proposed metrics include the diversity of skill sets and the number of teams that have been able to receive financial support or start a business after the event.

In our study, to measure the impact of a hackathon, we used the metrics of (1) growth of the digital health community and (2) subsequent events (eg, hackathons) that followed the initial hackathon. The former can be easily measured by creating mailing lists or social media groups. These metrics could reflect the impact of the hackathon better than the immediate results in the form of prototypes developed by the participants.

### Hackathon Team Building and Flow

Generally, team building at hackathons is a delicate topic. Firstly, an ideal team should consist of several disciplines, including developers, designers, entrepreneurs, health care professionals, and patients [7]. Secondly, the optimal size of a team is crucial. Based on studies of problem solving in groups, a total number of 5 people is recommended [17], whereas some hackathons allow larger team sizes of up to 8 people per team [16,18]. Lastly, a team may be formed by the organizers or by the participants themselves prior to or at the hackathon. We encourage organizers to define multidisciplinary teams of 5-7 participants in advance, respecting individual needs and backgrounds regarding the proposed challenge and team composition.

We divided the hackathon flow into the following steps: team up, brainstorm, hack, prepare pitch, and present. Using a systems approach, which was developed by MIT Hacking Medicine, the steps *brainstorm* and *hack* can be further divided into four phases: identification of the problem, description from the perspective of different health care actors, alteration as a solution approach, and implementation for early user feedback [19]. The advantage of using the systems approach at a hackathon may be that it follows a structured path to product development, forcing the participants to identify relevant pain points, highlighting it from different perspectives, and focusing the final pitch toward the implementation of the new solution into clinical routine. A project template to be filled in by the participants prior to the hackathon may be useful and may shorten the two phases of identification and description, leaving more time for transdisciplinary work on the solution approach.

### Recommendations for Future Hackathons

In this work, we developed systematic recommendations for organizing a small hackathon with minimal effort and resources. A major difference in comparison to larger hackathons is the reduced number of logistic challenges, giving the organizers more flexibility. In regard to timing, the actual planning for the hackathon may begin only 3 months prior to the event for smaller hackathons, compared to larger ones where planning has to start 6 months in advance [7,14].

Other available recommendations for hackathons include the extended hackathon model [16]. Wang et al developed this model over a course of eight hackathons by including seminars and workshops about design thinking, hardware prototyping, or business plan development into a weekend hackathon. Notably, the number of participants was also rather small per event, ranging from 18 to 55 participants per hackathon. However, the overall size of this hackathon series could discourage potential academic hospitals from conducting transdisciplinary events themselves, due to the high effort involved.

### Limitations

Our study is limited by the choice of the methodology and is possibly biased by the fact that most of the researchers were also involved in conducting the hackathon. The systematic recommendations were developed in a transdisciplinary approach based on three hackathons. Further hackathons applying these recommendations should be used to validate stated findings. To increase the significance, interview protocols or survey results from hackathon participants should be included in future research.

### Conclusions

A hackathon may break down the barriers between technical experts—who are able to build innovative technologies—and clinicians or patients—who know best which solution is sustainable—by physically bringing both groups together in one space and closing gaps in language and character.

With this study we were able to show that small hackathons are an effective way to bring innovation to health care and may be more cost- and time-efficient in the long run than larger hackathons. The systematic recommendations are useful for everyone who wants to bring innovation and a fresh breeze into the rigid structures of academic hospitals through transdisciplinary hackathons.

## Abbreviations

BIH: Berlin Institute of Health
CAMTech: Consortium for Affordable Medical Technologies
DFG: German Research Foundation
MIT: Massachusetts Institute of Technology
